# HIV-1 and Its Resistance to Peptidic Carbohydrate-Binding Agents (CBAs): An Overview

**DOI:** 10.3390/molecules191221085

**Published:** 2014-12-15

**Authors:** Geoffrey Férir, Stephanie C. Gordts, Dominique Schols

**Affiliations:** Laboratory of Virology and Chemotherapy, Rega Institute for Medical Research, University of Leuven, Minderbroedersstraat 10. Leuven B-3000, Belgium; E-Mails: Geoffrey.ferir@rega.kuleuven.be (G.F.); Stephanie.gordts@rega.kuleuven.be (S.C.G.)

**Keywords:** HIV, carbohydrate-binding agents, resistance, therapy, microbicide, *N*-linked glycans, deletions

## Abstract

The glycoproteins on the surfaces of enveloped viruses, such as HIV, can be considered as a unique target for antiviral therapy. Different carbohydrate-binding agents (CBAs) target specific glycans present on viral glycoproteins of enveloped viruses. It has been shown that long-term CBA pressure *in vitro* can result in mutant HIV-1 isolates with several *N*-linked glycan deletions on gp120. These studies demonstrated that mainly high-mannose type glycans are deleted. However, interestingly, N241, N262 and N356 on gp120 have never been found to be affected after prolonged CBA exposure. Here, we review the mutation and (cross)-resistance profiles of eleven specific generated CBA-resistant HIV-1 strains. We observed that the broad-neutralizing anti-carbohydrate binding mAb 2G12 became completely inactive against all the generated CBA-resistant HIV-1 clade B isolates. In addition, all of the CBAs discussed in this review, with the exception of NICTABA, interfered with the binding of 2G12 mAb to gp120 expressed on HIV-1-infected T cells. The cross-resistance profiles of mutant HIV-1 strains are varying from increased susceptibility to very high resistance levels, even among different classes of CBAs with dissimilar sugar specificities or binding moieties [e.g., α(1,3), α(1,2), α(1,6)]. Recent studies demonstrated promising results in non-topical formulations (e.g., intranasally or subcutaneously), highlighting their potential for prevention (microbicides) and antiviral therapy.

## 1. Introduction

CD4^+^ immune cells are the main target cells of human immunodeficiency virus (HIV), the etiological agent of acquired immune deficiency syndrome (AIDS). Infection starts when the viral envelope glycoproteins gp120 interact with the cellular CD4 receptors, present on T helper cells (Th cells), dendritic cells (DCs), monocytes and macrophages. The initial gp120/CD4 interaction induces a first set of conformational changes inside gp120 in order to gain subsequent binding with the cellular chemokine receptors CCR5 or CXCR4 for, respectively, CCR5-tropic (or R5) and CXCR4-tropic (or X4) HIV-1 strains. Upon these coreceptor interactions, a second set of conformational changes induce the exposure of gp41, which initiates membrane fusion by the formation of the six-helix-bundle, in combination with the host cell endocytic machinery [1,2].

In this review, we focus on *N*-linked glycan structures, which are abundantly present on the surface of HIV-1 gp120. Gp120 is displayed as a trimer and each monomer consists of 5 variable regions (V1–V5) and 5 conserved domains (C1–C5), which are heavily glycosylated. These glycans account for ~50% of its molecular mass [3,4].

**Figure 1:**
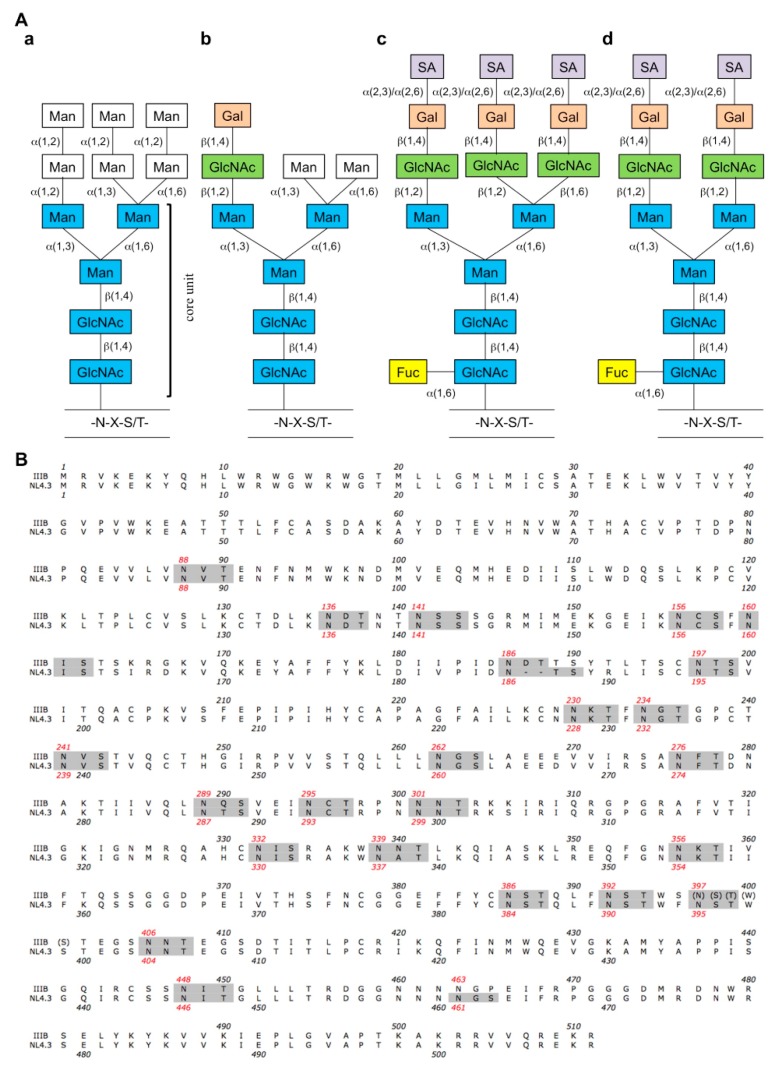
(**A**) Examples of *N*-linked glycans present on gp120. (**a**) Oligomannose or high-mannose (Man_9_) type glycans, (**b**) hybrid type glycans, (**c**) tri- and (**d**) bi-antennary complex type glycans. N is asparagine; X is any amino acid except proline; S is serine and T is threonine. The abbreviations for the sugar units are: Fuc is fucose; Gal is galactose; GlcNAc is N-acetylglucosamine; Man is mannose and SA is sialic acid. The common pentasaccharide core unit is also marked in blue; (**B**) HIV-1 gp120 amino acid sequence and glycosylation sites. Alignment of the gp120 envelope protein sequences of the T cell line adapted HIV-1 strains IIIB and NL4.3. The N-glycosylation sites are shown in grey and the sequon numbers in red. The N397 glycan is not present in the viral strain we used due to the NSTWS sequence deletion as indicated in brackets. The N463 glycan is only present as N461 in our laboratory HIV-1 NL4.3 wild-type strain and not in the sequenced HIV-1 IIIB wild-type strain.

**Figure 2:**
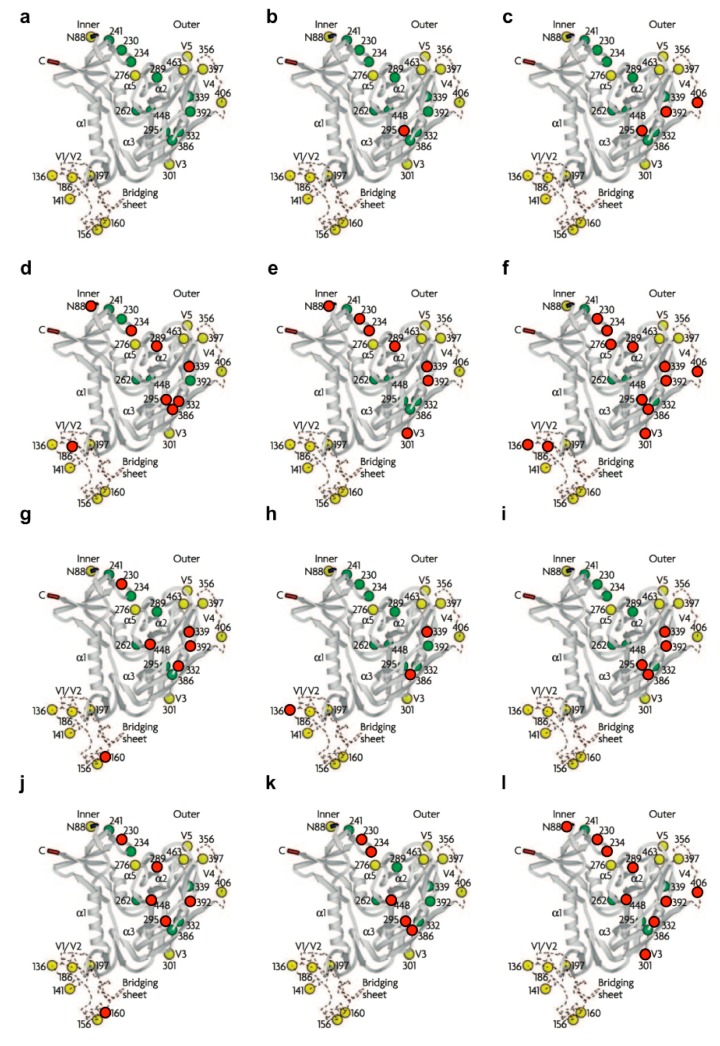
Positions of mutated *N*-linked glycans under selective CBA pressure. (**a**) Ribbon diagrams show the 24 *N*-linked glycosylation sites in recombinant (monomeric) gp120 of wild-type HIV-1 IIIB according to Leonard *et al.* [3] and Kwong *et al.* [11]. The green dots indicate the high-mannose type glycans and the yellow dots the hybrid/“potential” complex types. Recent findings by Doores *et al.* [8] and Bonomelli *et al.* [9] demonstrated that the glycan composition of recombinant monomeric gp120 differs greatly from that of trimeric gp120 present on infectious viral particles, of which the latter were used in the described resistance studies. Based on these data, the specific glycosylation assignments presented here are an overestimate of complex glycan content. Positions of the glycans deleted on gp120 under increasing concentrations of the following CBAs: (**b**) 2G12 mAb (NL4.3), (**c**) 2G12 mAb (IIIB), (**d**) HHA (IIIB), (**e**) GNA (IIIB), (**f**) AH (IIIB), (**g**) CV-N (IIIB), (**h**) CV-N (NL4.3), (**i**) MVN (NL4.3), (**j**) BanLec (IIIB), (**k**) GRFT (IIIB) and (**l**) UDA (IIIB) are marked with red dots. Figure 2a, reproduced with permission, from reference [12].

The synthesis of these glycans is beyond the scope of this review (see reference [5]). As the degree of glycosylation depends on the HIV subtypes and the host cell machinery, the number of glycosylation sites vary between 18 and 33. Structural characterization of the carbohydrates on gp120 by Mizuochi and coworkers indicated that the glycoprotein is unique in its degree of diversity of oligosaccharide structures, as it contains 33% high-mannose type, 4% hybrid type and 63% of complex type sugars (Figure 1A) [6,7]. These complex types, which are more than 90% fucosylated and/or sialylated, could be further subdivided in 4 categories: mono- (~2%), bi- (~38%), tri- (~12%) and tetra-antennary (~10%) sugars. Leonard and colleagues studied the glycan structures on gp120 of HIV-1 strain IIIB and found 24 putative *N*-linked glycosylation sites (Table 1, Figure 1B and Figure 2a). These sites are characterized by the sequon “N-X-S/T” (Asn-X-Ser/Thr) in the amino acid sequence of gp120, with X being any amino acid except proline (Figure 1). Eleven oligomannose (high-mannose) and/or hybrid type glycans were found and 13 of the complex type [3]. All have a common pentasaccharide core unit (Man_3_GlcNAc_2_) linked to the amide side chain of asparagine (Figure 1A). However, the abovementioned percentages of various glycan types were derived from an analysis of recombinant monomeric gp120 [3,6,7]. Recent studies indicated that the glycan composition of trimeric gp120 on replication competent HIV virions (and pseudoviral particles) contains predominantly oligomannose glycans, independently of the cellular production system (e.g., PBMCs or cultured human HEK293T cells) and viral subtypes (e.g., subtypes A, B and C) [8,9]. As the HIV-1 resistance studies described in this review were performed in HIV-1 replication assays, the site-specific glycosylation assignments presented here most likely overestimate the complex glycan content. We therefore renamed them “potential” complex glycans (Table 1). Remarkably, despite using the host cell glycosylation machinery, the glycans on the HIV envelope glycoproteins are predominantly high-mannoses in contrast to the host cell glycoproteins [10].

**Table 1:** Mutations in HIV-1 gp120 appearing under selective CBA pressure in T cell cultures.

Sequon ^a^	N-glycan	NL4.3^2G12res.^	IIIB^2G12res.^	IIIB^HHAres.^	IIIB^GNAres.^	IIIB^AHres.^	IIIB^CV−Nres.^	NL4.3^CV−Nres.^	NL4.3^MVNres.^	IIIB^BanLecres.^	IIIB^GRFTres.^	IIIB^UDAres.^
NVT (N88)	“potential” complex			x	x							x
NDT (N136)	“potential” complex					x		x				
NSS (N141)	“potential” complex									(NG) ^d^		
NCS (N156)	“potential” complex											
NIS (N160)	“potential” complex						x			x		
NDT (N186)	“potential” complex			x		x ^e^						
NTS (N197)	“potential” complex											
NKT (N230)	high-mannose				x	x ^e^	x			x	x	x
NGT (N234)	high-mannose			x	x	x					x	x
NVS (N241)	high-mannose											
NGS (N262)	high-mannose											
NFT (N276)	“potential” complex					x						
NQS (N289)	high-mannose			x	x	x ^e^				x		x
NCT (N295)	high-mannose	x	x	x		x			x	x	x	
NNT (N301)	“potential” complex				x	x						x
NIS (N332)	high-mannose			x			x					x
NNT (N339)	high-mannose			x	x	x	x	x	x			
NKT (N356)	“potential” complex											
NST (N386)	high-mannose			x		x ^e^		x	x		x	
NST (N392)	high-mannose		x		x	x ^e^	x		x	x		x
NST (N397) ^b^	“potential” complex											
NNT (N406)	“potential” complex		x			x ^b^						x
NIT (N448)	high-mannose						x			x	x	x
NES (N463) ^c^	“potential” complex										(NG) ^d^	
Total		1	3	8	7	12	6	3	4	6	5	9

^a^: Assignment of glycosylation sites according to Leonard *et al.* [3]; ^b^: The N397 glycan was not present in the wild-type IIIB strain throughout the AH resistance experiments due to the NSTWS sequence deletion as indicated in brackets in Figure 1B. Therefore, the N401 mutation found for the HIV-1 IIIB^AHres.^ strain corresponds to the N406 according to Leonard *et al.* [3]; ^c^: N463 is only present in the HIV-1 NL4.3 wild-type strain as N461 and not in the HIV-1 IIIB wild-type strain (1B); ^d^: (NG): new glycan created; ^e^: x are the *N*-linked glycan deletions used to calculate the fold-resistance for AH in Table 2.

**Figure 3:**
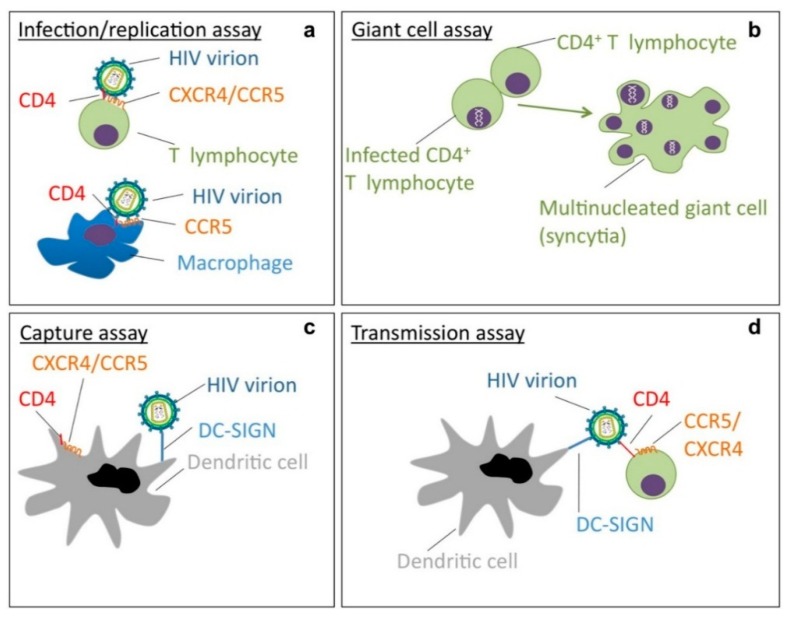
Mechanisms of action of CBAs endowed with anti-HIV acitivity. Four different well-described infection pathways important in HIV pathogenesis, which are all efficiently inhibited by CBAs: (**a**) HIV-1 infection/replication assay; (**b**) giant cell or cocultivation assay; (**c**) HIV-1 DC-SIGN capture assay and (**d**) DC-SIGN-related HIV-1 transmission assay.

These *N*-linked glycans are a unique target for the class of carbohydrate-binding agents (CBAs). CBAs can be found in various different species throughout nature such as prokaryotes, algae, plants and vertebrates [12]. They are characterized by a broad array of sugar specificities (such as mannose, glucose, fucose, N-acetylglucosamine or sialic acid). CBAs endowed with antiviral activity target HIV replication via four different pathways (Figure 3): (*i*) They inhibit entry of free HIV particles in PBMCs, monocyte/macrophages and DCs (Figure 3a); (*ii*) they block the formation of multinucleated giant cells or syncytia, formed after fusion between persistently HIV-infected T cells and CD4^+^ non-infected T cells (Figure 3b); (*iii*) they prevent the capture of HIV virions by DC-SIGN (Dendritic Cell-Specific Intercellular adhesion molecule 3 Grabbing Non-integrin) (Figure 3c) and (*iv*) block subsequent HIV transmission to uninfected CD4^+^ target T cells (Figure 3d) [12]. DC-SIGN, expressed on DCs and macrophages, acts as a tetrameric attachment receptor for many sorts of pathogens (e.g., viruses, bacteria, parasites; see reference [10] and references therein for more information). DC-SIGN has specificity for mannose and fucose-containing glycans [13,14], which are, as described above, abundantly present on gp120 of HIV particles. As HIV is able to hijack the endocytic function of DCs, this viral capture is involved in the dissemination of HIV towards the lymph nodes and highlights the ingenious strategies used by HIV for its transfer to infect vulnerable CD4^+^ T cells [15]. The recent insights that replication competent HIV virions, in contrast to recombinant gp120, contain more oligomannose glycans render the mannose-targeting CBAs as ideal potential candidates for antiviral purposes.

Prolonged exposure of HIV to CBAs can result in the appearance of mutations in the sequon motifs on gp120 that render them resistant to CBAs. This evokes *N*-linked glycan deletions, which creates holes in the protective glycan shield and could trigger cellular and humoral immune responses. In addition, those mutations can also affect the viral fitness and infectivity [12]. Here, we will overview and discuss the resistance mutations that appear under the pressure of eleven members of this class of CBAs: 2G12 mAb, *Hippeastrum hybrid* agglutinin (HHA), *Galanthus nivalis* agglutinin (GNA), Actinohivin (AH), Cyanovirin-N (CV-N), Microvirin (MVN), Banana lectin (BanLec), Griffithsin (GRFT), *Oscillatoria aghardii* agglutinin (OAA), *Urtica dioica* agglutinin (UDA) and *Nicotiana tabacum* agglutinin (NICTABA). We also highlight their cross-resistance patterns and their inhibitory effects on 2G12 mAb binding to gp120.

## 2. Resistance and Cross-Resistance Pattern of CBAs with Potent Anti-HIV Activity

### 2.1. Mannose-Specific CBAs

#### 2.1.1. Monoclonal Antibody 2G12

In the mid-nineties, the broad-neutralizing 2G12 mAb has been isolated from the blood of an HIV-infected individual [16] and follow-up studies showed a high affinity for man-α(1,2)man-linked sugars of high-mannose type glycans on the silent face of gp120 around the C4/V4 region [17,18], which is actually a poorly immunogenic region [11,19]. Binding inhibition of 2G12 mAb to gp120 was observed in the presence of mannose and not glucose, GlcNAc or galactose [18]. MAb 2G12 has a unique structure as Calarese and coworkers demonstrated, based on crystal structures of Fab 2G12 with unliganded Fab and its complexes with Man_9_GlcNAc_2_ or the disaccharide man-α(1,2)man, that the two Fab domains of 2G12 assemble into an interlocked VH domain-swapped dimer [20].

Antiviral activity studies showed that 2G12 mAb broadly neutralized the majority of subtype B viruses (clinical isolates and laboratory strains) and in addition viruses of the non-subtype B group [17,21]. In our lab, we found that 2G12 mAb has rather limited broad-neutralizing activity, as it inhibits only clinical isolates of subtypes A and B and subtype B laboratory strains in the lower µg/mL-range (50% inhibitory concentration or IC_50_: 0.04–1.4 µg/mL) [22]. No antiviral activity (IC_50_ >20 µg/mL) was observed against group O HIV-1 strains and HIV-2 [22,23].

Increased 2G12 mAb pressure resulted quickly in HIV-1 resistant strains. Within three weeks (six passages), the HIV-1 NL4.3^2G12res^ virus showed one pure mutation (N295K) at position N295 (Table 1; Figure 2b), resulting in a decreased antiviral activity of >50-fold (IC_50_ >50 µg/mL; Table 2) [22]. In addition, the generated HIV-1 IIIB^2G12res^ strain developed three mutations in gp120 at N-glycosylation motifs: T297T/I (N295), T394T/I (N392) and T408T/I (N406) (Table 1; Figure 2c). These mutations resulted also in a complete loss of antiviral activity (IC_50_ >50 µg/mL) (Table 2) [22]. These findings are in agreement with previously published epitope mapping studies. Alanine scan mutagenesis experiments showed a significant decreased 2G12 mAb binding to gp120 after elimination of the asparagines at position N295, N332, N339, N386 and N392. More in depth studies indicated that N295, N332 and N392 are crucial for interaction with gp120 and withdrawal of the N339 and N386 glycosylations will produce more conformational perturbations or protein misfolding, indicating them as less critical for antibody binding [18,24]. Many clade C HIV-1 strains are resistant to 2G12 mAb neutralization [22,25], however introduction of N295 glycosylation site restored mAb binding [25].

**Table 2:** Resistance and cross-resistance profile of CBAs of *in vitro* generated CBA-resistant HIV-1 strains.

CBAs	NL4.3^2G12res^	IIIB^2G12res^	IIIB^HHAres^	IIIB^GNAres^	IIIB^AHres^	IIIB^CV−Nres^	NL4.3^CV−Nres^	NL4.3^MVNres^	IIIB^BanLecres^	IIIB^GRFTres^	IIIB^UDAres^
AH	N.D. ^a^	N.D.	N.D.	N.D.	19 ^b^	N.D.	N.D.	N.D.	>66	118	N.D.
MVN	2	N.D.	N.D.	N.D.	N.D.	N.D.	N.D.	>100	N.D.	4	N.D.
2G12 mAb	>50	>36	>15	>15	>25	>45	>45	>38	>25	>35	>29
HHA	12	2	919	490	1	17	4	3	3	5	117
GNA	6	1	926	581	N.D.	23	1	2	N.D.	N.D.	39
BanLec	1	N.D.	N.D.	N.D.	N.D.	N.D.	N.D.	5	24	13	N.D.
GRFT	10	N.D.	N.D.	N.D.	N.D.	N.D.	N.D.	14	>106	>1900	N.D.
CV-N	1	3	29	11	N.D.	20	7	4	N.D.	N.D.	83
OAA	3	N.D.	N.D.	N.D.	N.D.	N.D.	N.D.	2	2	2	N.D.
UDA	8	N.D.	N.D.	N.D.	1	1	3	3	1	3	24

*Notes*: ^a^: N.D. Not determined; ^b^: Legend fold resistance: the indicated colors describe the antiviral activity [more- (shown in blue) or less active (shown in green-yellow-orange and red)] of various CBAs against different *in vitro* generated CBA-resistant HIV-1 strains.
Increased sensitivity1 (none)≥2–9 (low)≥10–24 (moderate)≥25–49 (high)≥50 (very high)

The HIV-1 NL4.3^2G12res^ isolate showed an increased (three to 12-fold) susceptibility to the CBAs OAA, HHA, GNA and UDA (Table 2) [22,26,27]. CV-N and BanLec fully kept their inhibitory activity [23]. This 2G12-resistant virus demonstrated low to moderate levels of cross-resistance with, respectively, MVN (2-fold) and GRFT (10-fold) [23,26]. Remarkably, only the NL4.3^2G12res^ HIV-1 strain showed increased sensitivity to certain CBAs. Such observations were not made using the IIIB^2G12res^ HIV-1 isolate (Table 2; Figure 2b,c), which could indicate cell line- and/or viral-dependent effects or an ideal structural conformation for certain CBAs when only N295 was deleted on gp120 [22].

#### 2.1.2. Hippeastrum Hybrid Agglutinin (HHA)

HHA was isolated from the bulbs of *Amaryllis* and exists as a tetrameric protein. It has a total molecular weight of 50 kDa and showed high specificity for α(1,3) and α(1,6)-mannoses [28]. HHA demonstrated a very potent antiretroviral activity in replication and transmission assays against HIV-1 and HIV-2 in various cell lines (e.g., CEM cells, MT-4, PBMCs and macrophages) with IC_50_s ranging from 1.8 to 980 nM [22,23,26,29,30,31,32,33,34,35].

Our laboratory generated a HIV-1 IIIB^HHAres^ strain in CEM cell cultures. Up to 90 subcultivations were required to replicate in the presence of 500 µg/mL HHA. Prolonged HHA exposure resulted in the loss of eight *N*-linked glycans in gp120 due to the following mutations: T90I (N88), T188N (N186), T236A (N234), N289K (N289), T297I (N295), S334N (N332), T341I (N339) and N386D (N386) (Table 1; Figure 2d). This led to a >900-fold increase in IC_50_-values from 4.2 ± 2.4 nM (wild-type IIIB strain) to 3.9 ± 1.9 µM (HIV-1 IIIB^HHAres^ strain) (Table 2) [35]. Unfortunately, the HHA-resistant variant was found to be approximately 10-fold more infectious than the wild-type virus in CEM cells; the cell line in which the resistant virus was generated [35]. A similar degree of increased infectivity was also observed in the HIV-1 susceptible astroglioma transfected cell line U87.CD4.CXCR4 [35]. Moreover, this HIV-1 HHA-resistant IIIB strain showed moderate to very high levels of resistance against GNA (>900-fold), 2G12 mAb (>15-fold) and CV-N (29-fold). MAb 2G12 lost once more its activity completely (IC_50_ >25 µg/mL) [35].

#### 2.1.3. Galanthus Nivalis Agglutinin (GNA)

GNA was isolated from the bulbs of *Galanthus nivalis* (snowdrop). It exists, like HHA, as a tetrameric protein with a molecular weight of 50 kDa and has specificity for α(1,3)mannoses [36]. GNA has a broad-spectrum antiretroviral activity in replication and cell-to-cell transmission assays (IC_50_: 1.7–540 nM) [22,23,26,30,31,32,33,34,35]. No to weak anti-HIV activity was observed against HIV-1 subtype C strain ETH2220 and subtype G strain BCF-DIOUM with IC_50_s ≥ 2 µM [22,35].

The HIV-1 IIIB^GNAres^ strain was also selected in CEM T cells [35]. It took up to 70 passages to generate a resistant isolate that could replicate in the presence of 500 µg/mL of GNA. Mutation analysis of gp120 revealed that seven *N*-linked glycosylation sites were affected under GNA pressure: T90T/I (N88); T232M (N230); N234K (N234); N289N/D and/or S291S/F (N289); N301Y (N301); T341I (N339) and T394I (N392) (Table 1 and Figure 2(e)). This resulted in a >500-fold drug resistance profile as the IC_50_ increased from 5.4 ± 1.2 nM (wild-type IIIB) to 3.1 ± 2 µM (HIV-1 IIIB^GNAres^ isolate). The resistant strain demonstrated also an increased viral fitness (approximately 30-fold) compared to wild-type IIIB in CEM and U87.CD4.CXCR4 cell cultures [35].

The GNA-resistant isolate proved to be highly resistant to HHA (490-fold) and moderate to CV-N (11-fold). MAb 2G12 became completely inactive (IC_50_ >25 µg/mL) and demonstrated a resistance profile of >15-fold (Table 2) [35].

#### 2.1.4. Actinohivin (AH)

AH (12.5 kDa) has been isolated from the actinomycete *Longispora albida* K97-0003^T^ [37]. AH contains 114 amino acids and consists of three segments, each containing one sugar-binding pocket, as observed by 3D analysis [38]. It exhibits a strong and highly specific affinity for gp120 and demonstrates very potent anti-HIV activity in replication and cocultivation assays in the lower nM-range with IC_50_s varying from 25 to 1200 nM [29,37]. However, its anti-HIV activity can be greatly improved through dimerization [39]. Various studies demonstrated that AH has specificity for man-α(1,2)man structures and pointed which amino acids are essential for its anti-HIV activity [29,40]. The binding of AH to gp120 can be inhibited by yeast mannan [41] and only by [α(1,2)man]_3_ oligosaccharides, indicating no affinity for α1,3/α1,6-mannose or GlcNAc-based sugars [29].

Exposure of the HIV-1 strain IIIB in CEM cell cultures to increasing concentrations of AH, resulted in the loss of up to 12 different *N*-linked glycans (Table 1) [29]. The following resistance mutations were found in the sequons of various sequenced resistant HIV-1 isolates: T138T/I (N136); T188N/T (N186); T232T/M, T232K/T, T232K and T232M (N230); N234K/N (N234); N276N/I (N276); N289N/S, N289N/Y, N289Y, S291S/F, S291V/F, S291F (N289); T297T/I, T297T/A, T297A (N295); T303T/I (N301); N339K/N, N339N/D (N339); N386K/N (N386); T394T/I, T394I (N392); T403T/I (N401) (Figure 2(f)). Surprisingly, a *N*-linked glycosylation was also created at position 29 [29]. The highest degree of AH resistance (19-fold) was observed with the following five mutations appearing simultaneously in gp120: T188N/T (N186), T232K/T (N230), N289N/Y (N289), N386K/N (N386) and T394T/I (N392), as its antiviral activity decreased from 25 ± 5 nM (wild-type HIV-1 IIIB) to 480 ± 89 nM (HIV-1 IIIB^AHres^ strain) (Table 1 and Table 2) [29].

Cross-resistance studies demonstrated that the wild-type IIIB and generated HIV-1 IIIB^AHres^ strain were equally susceptible to the plant lectins HHA and UDA, while 2G12 mAb lost >25-fold of its activity (Table 2), and was thus considered inactive [29].

#### 2.1.5. Cyanovirin-N (CV-N)

CV-N is an 11 kDa protein isolated from the blue-green algae *Nostoc ellipsosporum* [42]. It consists of 101 amino acids and structure analysis indicated that CV-N exists a monomer [43]. However, Yang *et al.* reported crystal structures that contain domain swapped CV-N dimers [44]. Later studies demonstrated that CV-N can exist in solution in both monomeric and dimeric form [45]. Four sugar-binding domains can be found in the dimeric form. Various studies pointed out that CV-N has a high affinity for high-mannose glycans with eight or nine mannose residues and that it recognizes the man α(1,2)-man moieties [46,47,48].

CV-N demonstrated a very potent and consistent anti-HIV-1 and anti-HIV-2 activity against T cell line adapted strains and clinical isolates in cell cultures with IC_50_s varying between 0.1 and 164 nM [22,26,34,42,49,50].

CV-N resistance studies were performed using HIV-1 strains IIIB and NL4.3 with gradually increasing concentrations of CV-N ranging from 0.1 µg/mL (~9 nM) to 2 µg/mL (~180 nM), due to cytotoxicity problems at concentrations >2 µg/mL in CEM cell cultures the dosage could not be further increased. A total of seven virus isolates were then sequenced for *N*-linked glycan deletions [49]. Here, we will focus on mutant strains showing the highest levels of resistance to CV-N. Six N-glycosylation sites were found to be deleted in HIV-1 IIIB^CV−Nres^ virus based on the following observed mutations: S162S/N (N160); T232T/M (N230); S334N (N332); N339S (N339); N392N/T (N392) and N448S (N448). Two mutations were observed in HIV-1 NL4.3^CV−Nres^ isolate: T341A (N339) and N386D (N386). One isolate in the NL4.3 resistant arm showed a mutation at position N136 (N136N/K), which intriguingly disappeared at higher concentrations. Overall, these data indicate that up to eight *N*-linked glycans can be deleted in the presence of increasing concentrations of CV-N (Table 1, Figure 2g–h). A 20-fold resistance ratio was observed for CV-N in the IIIB selection arm, as the IC_50_ increased from 2 ± 1 nM (wild-type IIIB) to 40 ± 21 nM (IIIB^CV−Nres^ strain) [49]. These data indicated that the combined deletion of N230, N392 and N448 resulted in a high level of CV-N resistance [49]. The NL4.3^CV−Nres^ isolate evoked only a 7-fold decrease in antiviral activity (IC_50_: 0.7 ± 0.5 nM [wild-type]; 5 ± 0 nM [CV-N-resistant strain]) [49]. Around the same period, a comparable study by Hu *et al.* in C8166 cells using HIV-1 IIIB, resulted in a CV-N-strain with five glycans of the high-mannose type removed at positions N289, N332, N339, N392 and N448, resulting in a 24-fold increase of IC_50_-values [51]. Four of the abovementioned sequon mutations were also described in our laboratory [49], however Hu and coworkers did not observe the mutation at position N230 and according to their mutagenesis study the loss of N230 did not affect the antiviral activity of CV-N [51].

As shown in Table 2, 2G12 mAb completely lost its antiviral activity (IC_50_ >50 µg/mL; >45-fold) against both generated CV-N-resistant strains, while the GlcNAc-specific lectin UDA lost no more than a 3-fold of its inhibitory activity. The HIV-1 IIIB^CV−Nres^ strain was, respectively, 17-fold and 23-fold less susceptible to the plant lectins HHA and GNA [49]. The CV-N-resistant isolate from Hu and colleagues, showed cross-resistance against GNA (21-fold) and GRFT (20-fold). MAb 2G12 lost completely its antiviral activity (IC_50_ >40 µg/mL) [51]. Minor differences in cross-resistance were seen with the NL4.3-resistant CV-N variant (Table 2), which can be explained by the different sugar deletions throughout gp120.

#### 2.1.6. Microvirin (MVN)

MVN (14.3 kDa) was isolated from the cyanobacterium *Microcystis aeruginosa* PCC7806 and shows 33% identity with cyanovirin-N (CV-N) [52]. MVN consists of 108 amino acids and has a monomeric form in solution. Only one carbohydrate-recognition site is present, which shows specificity for man-α(1,2)man moieties [52,53]. MVN demonstrated a broad-spectrum anti-HIV-1 activity in PBMC cultures (IC_50_s: 2.1–167 nM) with no cytotoxicity. Comparable activity was also observed in giant cell formation- and DC-SIGN-mediated transmission assays (IC_50_s: 125 nM–189 nM) [26].

MVN resistance studies using HIV-1 NL4.3 in MT-4 cells resulted in four mutations in *N*-linked glycosylation motifs of gp120 at positions: T297I (N295); T341T/I (N339); N386K/N (N386) and N392D (N392) (Table 1 and Figure 2i) [26]. According to these deletions, the IC_50_ of MVN increased >100-fold from 5.6 ± 1.4 nM (wild-type HIV-1 NL4.3) to 576 ± 84 nM (HIV-1 NL4.3^MVNres^ virus) (Table 2).

The MVN-resistant isolate showed a three to 4-fold degree of resistance against CV-N, HHA and UDA. No antiviral activity could be observed for MAb 2G12 (IC_50_ >50 µg/mL; >38-fold resistance). In contrast, the isolate became a 2-fold more susceptible for the plant lectin GNA (Table 2) [26]. Later studies showed that this resistant virus, compared to the wild-type NL4.3 strain, was also two to 5-fold more susceptible to other CBAs such as OAA and BanLec, In addition, the MVN-resistant strain showed a 14-fold decreased susceptibility to GRFT (Table 2) [23,27].

#### 2.1.7. Banana Lectin (BanLec)

BanLec can be purified from the *Musa paradisiac* or *Musa acuminata* or in the leaves of intact plants after jasmonate treatment [54,55]. BanLec exists as a homodimeric protein with two subunits of 15 kDa. Cloning studies showed that it belongs to the group of mannose-specific jacalin-related lectins, and was found to be a T cell mitogen [54,55]. Each monomer has two carbohydrate-binding sites, of which the second was quite unique in the jacalin-related lectin family [56]. Sugar binding experiments indicated its specificity for high-mannose type glycans containing eight or nine mannose residues and no binding with Man_6_ or mono- and bi-antennary complex type glycans [54]. Later binding and crystal structure studies showed unique carbohydrate-binding properties to internal 3-*O*-α-d-glucopyranosyl units and α(1,3)-linked glucosyl residues as well as β(1,3)-linkages at the reducing termini [56,57,58].

Various *in vitro* replication and transmission assays demonstrated that BanLec has a very potent and consistent anti-HIV-1 and anti-HIV-2 activity (IC_50_: 0.13–9.7 nM) [23,30,59,60]. The antiviral activity of BanLec was more pronounced than its weak mitogenic activity observed in PBMC cultures [23].

In our lab, we generated a HIV-1 IIIB^BanLecres^ strain in C8166 cells. After 59 passages, six mutations were found in gp120: N160N/D (N160); N230N/T and T232T/M (N230); S291F (N289); T297I (N295); N392K (N392) and N448K/N (N448) (Table 1 and Figure 2j). At position 142 the serine to asparagine mutation (S142N/S) results in a new glycan, neighboring the existing N141 “potential” complex glycan (Figure 1B and Table 1) (unpublished data). Its function is not known, but it could be a compensatory glycan important for viral fitness. The resistance profile of this mutant virus against BanLec and various other CBAs was evaluated in MT-4 cells, TZM-bl cells and PBMCs. Pooled resistance data showed a 24-fold degree of resistance to BanLec (Table 2). Very high levels of cross-resistance of this generated BanLec-resistant virus were observed against the CBAs AH and GRFT (>66-fold). No antiviral activity was observed with MAb 2G12 (IC_50_ >20 µg/mL), while HHA and OAA remained active albeit to a lesser degree (two to 3-fold) (Table 2). No shift in susceptibility was observed for this resistant isolate to the GlcNAc-binding CBA UDA.

#### 2.1.8. Griffithsin (GRFT)

GRFT was originally isolated from the red algae *Griffithsia sp.* in the waters of New Zealand and exists as a domain swapped unique dimeric protein. Each monomer, which has a molecular weight of 12.7 kDa, consists of 121 amino acids and has three very similar carbohydrate-binding pockets [61,62]. Sugar-binding studies showed that the binding of GRFT to soluble gp120 was inhibited by glucose, mannose and GlcNAc [61]. Various crystal structures and modeling studies demonstrated an interaction of GRFT with high-type mannose (Man_9_GlcNAc_2_), mannose, GlcNAc, (1,6)α-mannobiose, glucose and maltose [62,63,64]. Monomeric forms of GRFT exhibited a strong reduction in its anti-HIV activity [65,66], which indicates that the anti-HIV activity of GRFT stems from cross-linking and aggregation of virions through multivalent interactions with the carbohydrates present on gp120.

It is the most potent anti-HIV CBA described to date with a consistent and broad-spectrum antiviral activity even in the pM to lower nM-range (IC_50_: 2 pM–56 nM) in infection/replication assays and different cell-to-cell transmission systems [23,60,61,67,68,69,70,71].

Resistance selection experiments resulted in five high-mannose *N*-linked glycan deletions in gp120 of HIV-1 IIIB^GRFTres^ strain developed in C8166 cell cultures: T232M (N230), T236I (N234), N295Y (N295), N386N/D and T388T/A (N386) and T450N (N448) (Table 1 and Figure 2k) [27]. A >1900-fold decrease in susceptibility for GRFT was observed with this mutant virus in MT-4 cells [IC_50_: 20.5 ± 3.8 pM (wild-type IIIB) to >39 nM (GRFT-resistant strain)]. The observed mutations are in accordance with the findings of other research groups, who indicated also the importance of N234, N295 and N448 for GRFT-resistance [68,69]. Moreover, long-term exposure of GRFT in our cell culture system induced a novel sequon at position N463 created by the proline to serine substitution at position 465 (P465S) (Table 1). The importance of this new glycan is not known, however, it should be noticed that this *N*-linked glycan is present in the original HIV-1 IIIB strain, according to Leonard and coworkers [3] and in the HIV-1 NL4.3 wild-type strain (Figure 1B). These data also highlight that the antiviral potency of CBAs does not impact the emergence of the number of mutations. The GRFT-mutant virus exhibited five mutations, while AH or HHA-resistant isolates obtained between eight and 12 mutations, all of them resulting in *N*-linked glycan deletions (Table 1). These observations indicate that the interaction kinetics with gp120 (and perhaps gp41) and the multivalent interactions with the *N*-linked glycans are the most important factors involved in viral resistance.

As indicated in Table 2, antiviral studies using the HIV-1 IIIB^GRFTres^ strain in MT-4- and TZM-bl cells demonstrated high levels of cross-resistance against AH (118-fold). This GRFT-resistant strain showed also low to moderate resistance against MVN (4-fold), UDA (3-fold), OAA (2-fold) and BanLec (13-fold). The anti-carbohydrate-binding mAb 2G12 demonstrated no antiviral activity (IC_50_ > 25 µg/mL) ([27] and unpublished data). Huang *et al.* mentioned that removal of N295 and N448 on gp120 renders HIV-1 highly resistant to GRFT, but maintained its sensitivity to CV-N and GNA [68]. Our GRFT-resistant isolate became 5-fold more sensitive to HHA. A comparable trend towards HHA was also observed using the NL4.3^2G12res^ HIV-1 strain (Table 2).

#### 2.1.9. Oscillatoria Agardhii Agglutinin (OAA)

OAA was isolated from the cyanobacterium *Oscillatoria agardhii* strain NIES-204 [72,73]. It has a molecular weight of 13.9 kDa and recognizes, as opposed to all other CBAs, the α3,α6-mannopentaose core of Man_8/9_ [74]. Recently, we could demonstrate that OAA has very consistent broad-spectrum anti-HIV activity in the lower nM-range (IC_50_: 19–60 nM) in several replication and transmission assays [27].

As OAA targets the branched central core unit of Man_8/9_, the HIV-1 strains resistant against MVN, BanLec and GRFT only displayed a 2-fold decrease in susceptibility for OAA (Table 2). As observed previously for the plant lectins HHA and GNA, the HIV-1 NL4.3^2G12res.^ isolate was found to be more susceptible for OAA (Table 2) [27]. Unfortunately, due to a low selectivity index (SI) in our cell culture system (SI ~13), we were not able to generate an NL4.3^OAAres^ HIV-1 strain (unpublished data).

### 2.2. N-Acetylglucosamine (GlcNAc)-Specific CBAs

#### 2.2.1. Urtica Dioica Agglutinin (UDA)

*Urtica dioica* agglutinin (UDA) is a monomeric GlcNAc-specific protein derived from the stinging nettle rhizomes. It has a total molecular weight of 8.5 kDa and contains two carbohydrate-binding sites with different affinities [75,76]. Structural modeling indicated that UDA efficiently interacts with the GlcNAc_2_Man_1_ residue, which is part of the core unit (Figure 1A) of glycans on gp120 [34].

UDA demonstrated a potent broad-spectrum anti-HIV-1 and anti-HIV-2 activity against T cell line adapted strains and clinical isolates in different cell lines (e.g., MT-4, CEM, PBMCs and macrophages) with IC_50_s ranging from 0.052 to 2.8 µM [30,31,34,77].

HIV-1 IIIB^UDAres^ virus was generated in CEM cell cultures, which took more than nine months (>90 passages) before the virus was able to replicate in the presence of 200 µg/mL of UDA [34]. Resistance mutations analysis showed that nine pure mutations occurred at *N*-linked glycosylation sites: T90I (N88), N230D (N230), T236I (N234), N289T (N289), N301Y (N301), S334N (N332), T394I (N392), N406Y (N406) and N448D (N448) (Table 1; Figure 2(l)). These deletions made the virus resistant as the IC_50_ shifts from 0.14 ± 0.04 µM (wild-type IIIB) to 3.3 ± 0.05 µM (UDA-resistant strain) (Table 2). The viral fitness of this resistant strain showed no marked differences with wild-type HIV-1 IIIB in CEM cells [34].

The mutant HIV-1 strain was highly resistant against HHA (117-fold) and CV-N (83-fold). The antiviral acitivity of GNA was a 39-fold reduced, while 2G12 mAb demonstrated no activity at all (IC_50_ >50 µg/mL) (Table 2) [34]. In addition, novel surface plasmon resonance (SPR) data showed that UDA binding to recombinant gp120 is also inhibited in the presence of certain mannoses, which demonstrates the broader sugar specificity of UDA than solely GlcNAc [78].

#### 2.2.2. Nicotiana Tabacum Agglutinin (NICTABA)

NICTABA is a non-glycosylated homodimeric protein of 38 kDa, which is expressed in the leaves of the tobacco plant after treatment with jasmonates [79,80]. It resides as a nucleocytoplasmatic plant lectin, where it is believed to be a signaling protein during stress physiology [81]. Glycan binding studies demonstrated that NICTABA has specificity for GlcNAc-oligomers on high-mannose and complex *N*-glycans [79,82]. Additional experiments using SPR technology indicate that NICTABA is a highly-specific GlcNAc binding lectin [78].

NICTABA has a very consistent anti-HIV activity in the lower nM-range (IC_50_: 5–230 nM) in different cell lines [30,78] and unfortunately, due to low selectivity indices (SI ~21) in cell cultures, we were not able to generate an HIV-1^Nicatabares^ strain (unpublished data). No cross-resistance data are available for NICTABA on CBA-resistant HIV-1 strains so far.

## 3. Inhibition of 2G12 mAb Binding to gp120 by CBAs

As mentioned above, Scanlan and coworkers demonstrated that the high mannose-type glycans N295, N332, N339, N386 and N392 are important for optimal 2G12 mAb binding [18]. Regarding Table 1 and Table 2, it seems quite obvious that the anti-carbohydrate binding mAb 2G12 possessed no longer antiviral activity against the generated CBA-resistant HIV-1 strains. Since for each of these strains, at least one of the mentioned high-mannose type *N*-linked glycans by Scanlan *et al.* [18], was deleted under selective prolonged CBA pressure.

In addition, Scanlan and coworkers also highlighted that CV-N was able to inhibit the binding of 2G12 mAb to gp120, but not *vice versa* [18]. Our data using MT-4/NL4.3 cells, correspond with these findings (Table 3). 

**Table 3:** CBAs inhibiting 2G12 mAb binding to gp120 expressed on HIV-1 NL4.3 infected T cells.

CBA	Binding Inhibition	IC_50_ (nM)	References
HHA ^a^	Yes	28–39	[22]/unpublished data
GNA ^a^	Yes	23–76	[22]
AH	Yes	93	[29]
CV-N ^a^	Yes	16–28	[22,26]
MVN	Yes	259	[26]
BanLec ^b^	Yes	4.5–14	[59]/unpublished data
GRFT ^b^	Yes	0.57	[61]/unpublished data
OAA	Yes	21	[27]
UDA	Yes	1000	[78]
NICTABA	No	>1000	[78]

^a^: data also obtained against HIV-1 strains IIIB, MN and NDK. ^b^: data from [59] and [61] were obtained against rgp120.

As further shown in Table 3, all of the investigated CBAs, with exception of NICTABA, inhibited dose-dependently the binding of 2G12 mAb to gp120 on NL4.3 infected MT-4 cells. The best 2G12 mAb binding inhibitors to gp120 were the mannose-specific lectins GRFT and BanLec with IC_50_s varying between 0.75 and 14 nM. These inhibitory data are in accordance with previously published observations of several binding studies [59,61]. The GlcNAc-binding CBA UDA demonstrated the weakest inhibitory profile (IC_50_: 1000 nM) (Table 3). In most cases, these differences in 2G12 mAb inhibitory binding potential correlated with their anti-HIV activity. As mentioned above, NICTABA is the only CBA that does not block the binding of 2G12 mAb to recombinant gp120 (IC_50_ >40 µg/mL or >1000 nM) (Table 3) [78]. Surprisingly, we could demonstrate that OAA also inhibited dose-dependently the binding of 2G12 mAb to gp120 (Table 3), which was quite unique, regarding the sugar specificity of this lectin. Overall, these data demonstrate that the majority of the anti-HIV CBAs target the 2G12 mAb epitope directly or alter the conformational epitope structure.

## 4. CBAs: A Role in HIV Prevention or Treatment?

By targeting viral transmission and entry/infection in four different pathways (Figure 3), as well as their high genetic barrier to resistance, the CBAs can be seen as a very promising class of agents in microbicidal applications (e.g., vaginal/rectal gels, creams, intravaginal ring systems) to prevent HIV infection in women and men [12].

The Centre for the AIDS Programme of Reseach in South-Africa (CAPRISA) 004 trial was the first breakthrough in the field of microbicidal research, which showed for the first time that HIV prevention was possible with a vaginal 1% tenofovir gel with two applications of treatment every 24 h [83]. However, as is done for HIV treatment, the prevention (or pre-exposure prophylaxis) strategy will also presumably comprise at least two antiretroviral agents. We could demonstrate that tenofovir (and other potent antiretroviral agents) showed synergistic to additive effects in different cell lines with various CBAs on viral transmission and on infection/replication of HIV strains having different envelope glycosylation patterns [30,60,70]. Remarkably, paired CBA combinations mainly showed synergistic activity against wild-type X4 and R5 HIV-1 strains [23,53]. The most surprising results were observed using HHA and GNA, which showed rather antagonistic to low additive effects on wild-type HIV-1 replication, but potent synergy against the 2G12 mAb-resistant and MVN-resistant HIV-1 strains. These data suggest that certain deleted *N*-linked glycans in the mutant gp120 proteins (Figure 1 and Figure 2) create holes and/or conformational changes on the surface, which result in better binding pockets for certain CBAs (such as HHA and GNA) to allow synergy. Such findings were in accordance with previously published data on increased activity of single CBA treatment on 2G12 mAb- and MVN-resistant strains (Table 2) [22,26]. It was also suggested that glycan deletions could render gp120 more susceptible to neutralizing antibodies [12]. Experiments with immunoglobulins and sera derived from HIV-1^+^ individuals showed that mutant viral envelopes were more sensitive than the wild-type [51]. Rhesus monkeys infected with mutant forms of simian immunodeficiency virus (SIV), having lost two glycosylation sites, produced more neutralizing antibodies, than the animals infected with parental virus [84].

A degree of synergy on antiviral activity with paired CBA combinations in HIV-1 replication assays, was also observed when the 2G12 mAb/HHA combination was evaluated in HIV-1 NL4.3 resistance experiments using C8166 T lymphoma cells. This CBA combination strategy markedly delayed drug resistance development and compromised viral fitness [85]. The anti-carbohydrate binding mAb 2G12 recognizes a specific epitope on gp120, which resulted very quickly in virus breakthrough [22]. Fast drug resistance development (≤5 passages) was likewise observed with members of the class of reverse transcriptase inhibitors (RTIs) (e.g., lamivudine) (data not shown). This process can be avoided by combining different antiretroviral agents, as used in highly active antiretroviral therapy (HAART). Other CBAs (e.g., HHA, GNA or GRFT) have multivalent interactions with the glycans on gp120 and create cross-links. Interesting observations described in this review were the inactivity of the 2G12 mAb against all evaluated CBA-resistant isolates (Table 2) and in addition all CBAs, with exception of NICTABA, interfered directly or indirectly with the 2G12 mAb binding epitope (Table 3).

A closer look at the mutations in the *in vitro* generated NL4.3^HHAres^ HIV-1 strain showed *N*-linked glycan deletions at two “potential” complex glycan sites (N160 and N463) and at four high-mannose sites (N230, N289; N339 and N386). The mutation in the sequon at N160 resulted in a new sequon at N162. Three of these mutations were also observed previously using CEM cells and HIV-1 strain IIIB: N289, N339 and N386, which were all high-type mannoses (Table 1) [35,85]. The NL4.3^2G12res^ HIV-1 strain had only one glycan deletion at position N392 [85]. This mutation again corresponds with previously published results with respect to 2G12 mAb resistance [18] and these observations indicate that comparable resistance mutations for 2G12 mAb appear in different cell lines under prolonged exposure [22,85]. When both CBAs were given simultaneously for longer periods to induce resistance, three mutations appeared in gp120 (N160, N339 and N386) and remarkably also one in gp41 (N672), together with the induced glycan at N162 [85]. The three mutations observed in HHA/2G12 mAb combination-arm, were also found in the single HHA-selected strain. It was assumed that HIV-1 exposure to paired CBA combinations with different binding sites will make it more difficult for the virus to escape drug pressure. This will result in marked decrease of resistance development and a lower mutation rate [85]. In depth cross-resistance studies demonstrated that the presence of this “potential” complex N-glycan site deletion in gp41, due to the asparagine to aspartate mutation at position N672 (N672D), resulted in higher levels of resistance against the CBAs AH, HHA and GNA compared to strains lacking this specific mutation [85]. As mentioned above, Huskens *et al.* [22] reported an increased susceptibility of the NL4.3 2G12mAb-resistant HIV-1 strain, carrying only the N295 mutation, for various CBAs (Table 2). Surprisingly, the NL4.3 variant, generated by Mathys and Balzarini, had also only one mutation, the N392 deletion, but showed no increased potency of CBAs [85]. The NL4.3^2G12res^ HIV-1 strain demonstrated solely resistance to 2G12 mAb (>57-fold), while the NL4.3^HHAres^ HIV-1 strain showed high levels (>35-fold) of (cross-)resistance against HHA, 2G12 mAb and GNA. Less than 10-fold cross-resistance was noticed against AH and UDA. The HIV-1 NL4.3^HHA/2G12res^ strain carrying also the N672D mutation showed cross-resistance against HHA (16-fold), GNA (10-fold), 2G12 mAb (>50-fold), AH and UDA (both 2-fold) [85]. Viral infectivity studies, as measured by p24 Ag levels, indicated a significant difference between the resistant (p24 Ag: ≤14 × 10^4^ pg/mL) and wild-type HIV-1 strains (p24 Ag: 30 × 10^4^ pg/mL). The HHA/2G12mAb mutated strains showed comparable amounts of p24 Ag concentrations as their single resistant strains [85].

CBA pressure selects HIV-1 resistant strains that have preferably high-mannose type N-linked glycans annihilated (Table 1). Of the six “potential” complex glycans sites (N136, N141, N156, N160, N186 and N197) present in the V1/V2 loop: the N141, N156 and N197 have never been mutated so far. However, our BanLec-resistant HIV-1 IIIB strain created a novel sequon at position 142 by mutating serine to asparagine (S142N) (Table 1; Figure 1B). A thorough investigation on the effect on glycan deletions on the V1/V2 loop of gp120 showed in some cases high compromised viral infectivity and replication rates, but an increased susceptibility of these mutated strains for certain CBAs such as the plant lectins HHA and UDA [86]. Positions assigned to be complex type glycans are most likely recognized by glycan-reactive broad-neutralizing antibodies with a documented complex-type glycan binding ability such as PG9 and PG16. These antibodies recognize Man_5_GlcNAc_2_ structure at position N160 and a sialylated complex *N*-linked glycan at position N156 [87]. The high-mannose glycan site N332 was found to be critical for the binding of PGT121. Glycan array and crystal structure studies also reported an interaction of this antibody with a putative V1/V2 bi-antennary complex glycan [88,89]. These data indicate also the necessity of the presence of complex glycans for optimal neutralizing activity by antibodies.

The N241, N262 and N356 sites on gp120 (which corresponds to N239, N260 and N354 in NL4.3 sequences [Figure 1B]) were never found to be mutated under prolonged CBA pressure (Table 1 and Figure 2) [90]; not even in the presence of two CBAs simultaneously [85]. Of these three glycans, the highly conserved N262 seems indispensable for viral entry and infectivity as shown by site-directed mutagenesis studies [51,90,91]. The N262Q mutation affects the folding and lysosomal degradation of gp120 [92]. Remarkably, the glycine at position 263 (or 261 for NL4.3) of this sequon (262NGS264) is also highly conserved among various HIV-1 strains and mutating this amino acid to an alanine results in a lower infectivity rate [90]. These findings demonstrate the importance of this highly conserved glycan and represent a potential “hot spot” for antiviral therapy.

A healthy vaginal microbiota, which is dominated by *Lactobacillus* species, play an important role in the protection of women for HIV transmission and infection by: (*i*) the direct/indirect production of antiviral components (e.g., bacteriocins, hydrogen peroxide and lactic acid), (*ii*) by stimulating the immune system and (*iii*) by lectin-mediated binding mechanisms [93]. This indicates that the class of CBAs may not harm the endogenous vaginal flora. Tsai and coworkers could clearly demonstrate that a CV-N gel protected 15 of 18 female macaques (*Macaca fascicularis*) from SHIV89.6P (simian/human immunodeficiency virus) infection after vaginal challenge [94]. They also found that CV-N was able to prevent rectal SHIV89.6P transmission in male macaques [95]. On the other hand, live recombinant *Lactobacilli* expressing CV-N could reduce vaginal SHIV transmission in macaques by 63% [96]. Later studies demonstrated that addition of these CV-N-expressing *Lactobacilli* neither changed the diversity of the vaginal microbiota, nor triggered pro-inflammatory changes to the local mucosa [97]. These data are very promising and suggest that *Lactobacilli* expressing CBAs can be used as “live-microbicides” to prevent HIV transmission and subsequent infections. In collaborative studies, we were able to express the CBAs AH and GRFT in certain *Lactobacilli* strains, however they could not be secreted due to unknown parameters (unpublished results). These data clearly indicate that not each CBA can be used as a “live-microbicide”. Each potential candidate should be thoroughly examined, as the class of CBAs are a diverse group of proteins with widely differing properties.

It is generally accepted that CBAs have a very low, if any, oral bioavailability. As described above, most *in vivo* research of antiviral active CBAs was performed in pre-exposure prophylaxis models [94,95]. More recently, different studies in murine models indicate that CBAs could also be used as antiviral therapy. A significant survival rate was observed when CV-N was given intranasally as prophylactic agent or at early initiation of treatment (within 6 h post-infection) in an influenza model [98]. Regarding the current Ebola virus outbreaks in several african countries (e.g., Liberia, Guinea and Sierra Leone) [99], the severity of Ebola infections in mice was shown to be reduced by CV-N treatment [100]. However, therapy with high doses of mannose-binding lectin (MBL), a component of the innate immune system, also diminished the Ebola infections in murine models [101]. Such findings open novel perspectives for the class of CBAs as even therapeutic agents. Beyond the scope of this review, but important to mention is that many CBAs have besides anti-HIV activity also a potent inactivating profile against other viruses, of which some are currently hard to treat or untreatable like Herpes simplex virus (HSV), Dengue virus, Ebola virus and/or Hepatitis C virus (HCV) [100,102,103,104]. However, their potent and mainly consistent antiviral activity, in this context against HIV, can sometimes be overshadowed by mitogenic and/or cell-agglutinating properties of certain CBAs.

CV-N is one of the most extensively studied CBA so far. Despite its potent broad antiviral activity, confusing data are found regarding toxicity and mitogenicity [42,49,50,105]. Buffa and colleagues found no cytoxicity of CV-N in PBMCs and cervical tissue explants (IC_50_ ≥ 0.45 mM). Low mitogenic activity was seen following three days of CV-N exposure, which could be greatly diminished when it was reduced to 2 h followed by three days of culture [50]. On the other hand, previous studies indicated pronounced mitogenic effects in PBMCs after pre-exposure of low sub-inhibitory CV-N concentrations, as increased viral replication values, compared to untreated conditions, were observed. This resulted in a higher susceptibility for R5 HIV-1 replication. In addition, increased levels of T cell activation markers [CD69 (early), CD25 (late) and HLA-DR (very late)] and of various pro-inflammatory cytokines were measured [49,105]. These potential side effects are probably unrelated to its carbohydrate-binding activity. Various other CBAs seem devoided of such cytotoxic and mitogenic properties. Safety studies using AH, HHA, GNA, UDA, GRFT and MVN demonstrated no upregulation of these T cell activation markers, nor significant induction of pro-inflammatory cytokines and chemokines [26,29,105,106]. In addition, GRFT, HHA and GNA did not show hemagglutination activity on human erythrocytes [32,107]. On the other hand, MVN can be a very good alternative for CV-N as microbicidal agent, as both molecules share 33% identity at the amino acid level [52]. MVN has a comparable anti-HIV-1 activity, with exception for HIV-2 and HIV-1 group O isolates. However, it is 50-fold less toxic and showed a much better safety profile than CV-N [26].

A thorough investigation also demonstrated that both GRFT and CV-N were able to interact with the cervical epithelial cells [106]. Distinct differences in their binding patterns were seen, as CV-N bound much more extensively throughout several layers of the cervical epithelium. The binding to the cell surface was significantly reduced when the sugar-binding sites were occupied with yeast mannan, while only partial reducing binding effects were seen for CV-N. This implies secondary binding mechanisms between CV-N and the cell surface. Remarkably, when the PBMCs were pre-treated for 24 h with GRFT or CV-N, subsequently washed and infected with R5 HIV-1 in the absence of these lectins, a potent inhibition of viral infection was observed for GRFT at a concentration of 0.13 nM, while CV-N at 182 nM significantly increased viral replication [26,106].

Follow-up safety studies by Barton and coworkers demonstrated that GRFT, when given subcutaneously to mice and guinea pigs, was present in concentrations well above its IC_50_-value and remained in plasma and serum for many days. In addition, GRFT accumulated in tissues like the spleen, kidney and liver. Organ weight investigation did suggest a nascent immune response to GRFT, but no antibodies were detected. GRFT did not alter animal behavior and no animals died due to treatment [107]. These optimistic data indicate that de-immunizing this molecule may be necessary for long-term treatment. At the moment, computational methods (e.g., EpiSweep) are available that produce designs of therapeutic proteins with preserved structural properties and minimal T cell epitopes [108,109], however further investigation of their production, antiviral and immunological properties are needed.

Overall, these data show that CBAs can have potential in the prophylaxis (microbicide) and treatment of (chronic) viral infections such as HIV.
